# Enquête entomologique dans le foyer historique de trypanosomose humaine africaine de Bendjé (Gabon)

**DOI:** 10.1051/parasite/2011184303

**Published:** 2011-11-15

**Authors:** L. Kohagne Tongué, R. Gounoue Kamkuimo, P. Mengue M’eyi, D. Kaba, F.J. Louis, R. Mimpfoundi

**Affiliations:** 1 Organisation de Coordination pour la lutte contre les Endémies en Afrique Centrale (OCEAC) Yaoundé Cameroun; 2 Laboratoire de biologie générale, Faculté des sciences – Université de Yaoundé I Yaoundé Cameroun; 3 Programme national de lutte contre la trypanosomiase humaine africaine – Ministère de la santé Gabon; 4 Institut Pierre Richet – Institut national de santé publique Abidjan Côte d’Ivoire

**Keywords:** glossine, trypanosomose humaine africaine, Gabon, glossina, human African trypanosomiasis, Gabon

## Abstract

La situation de la maladie du sommeil est très peu connue au Gabon. De nombreux foyers historiques n’ont pas été prospectés depuis plus de 15 ans. Le foyer historique de Bendjé fournit régulièrement quelques cas, dépistés passivement, qui concernent le plus souvent des pêcheurs dont il est *a priori* difficile de déterminer le lieu probable de contamination du fait de leur grande mobilité au cours de leurs activités. La présence des hommes infectés dans ce foyer historique pourrait favoriser son réveil s’il existe un contact étroit entre les différents éléments potentiellement présents du cycle épidémiologique (homme, vecteur, trypanosome). Afin de vérifier l’existence éventuelle d’un risque trypanosomien dans ce foyer, nous y avons mené une enquête entomologique. Des pièges ont été posés dans des biotopes fréquentés par l’homme et laissés en place pendant quatre jours. Trois espèces de glossines (*Glossina palpalis palpalis*, *G. pallicera newsteadi* et G. *caliginea*) ont été capturées et deux espèces de trypanosomes (*Trypanosoma vivax* et *T. brucei* s.l.) identifiées par PCR. Ces résultats suggèrent l’existence d’un cycle de transmission animal. Le contact entre les hommes et les glossines est particulièrement étroit dans tous les types de site prospectés, à l’exception de la mangrove.

## Abstract (en)

The situation of human African trypanosomiasis (sleeping sickness) is poorly known in Gabon. Most of the historical foci have not been investigated for more than 15 years. Few cases are passively recorded from the historical focus of Bendjé; they involved mainly fishermen but determining their contamination site is difficult because of their mobility due to their activity. The presence of these cases in that focus could favour its reactivation if the vector is still there. In order to assess a potential transmission risk in that area, an entomological survey has been carried out in it. Traps were set up during four days in different habitats used by humans during their daily activities. Three species of tsetse flies (*Glossina palpalis palpalis*, *G. pallicera newsteadi* and *G. caliginea*) were caught and two species of trypanosomes (*Trypanosoma vivax* and *T. brucei* s.l.) were identified by PCR. These results suggest the presence of an animal transmission cycle. Human-flies contact was confirmed in all type of habitats but no transmission was quantified in the mangrove.

## Introduction

La maladie du sommeil encore appelée trypanosomiase humaine africaine (THA) est endémique dans de nombreux pays d’Afrique centrale. Cette région fournit à elle seule 87 % du nombre total de malades diagnostiqués dans toute l’Afrique (WHO, 2005).

Au Gabon, la situation n’est pas bien connue. Depuis 2002, des malades sont régulièrement diagnostiqués dans la province de l’Estuaire, principalement dans le foyer de Komo-Mondah où sont diagnostiqués les trois quarts de malades déclarés par an dans le pays (Kohagne *et al.*, 2010). Cette région était déjà connue comme une zone active de THA au début du siècle dernier, ainsi que sous l’ère du Docteur Jamot (Millileri *et al*., 2009). De nombreux autres foyers historiques sont connus dans les provinces de l’Ogooué-Maritime, du Moyen-Ogooué, du Haut-Ogooué, de l’Ogooué- Ivindo, de la Nyanga et de la Ngounié, mais ces foyers n’ont pas été prospectés depuis plus de 15 ans, en raison de l’insuffisance de moyens financiers dont dispose le programme national de lutte contre la trypanosomiase humaine africaine de ce pays.

Dans la province de l’Ogooué-Maritime, des cas sporadiques de THA originaires du canton Océan sont diagnostiqués de manière passive par les structures sanitaires de la ville de Port-Gentil (chef-lieu de province) ; ce sont le plus souvent des pêcheurs diagnostiqués au stade tardif de la maladie et contaminés vraisemblablement au cours de leurs activités. Le canton Océan avait déjà été identifié en 1995 comme étant l’épicentre du foyer de Bendjé avec sept malades recensés (Amblard, 1996). Depuis lors, aucun dépistage actif n’y a plus été organisé. Ce canton est attenant au foyer actif de Komo-Mondah par voie fluvio-maritime et il est *a priori* difficile d’identifier un site particulier de contamination de ces pêcheurs malades qui parcourent tout le littoral gabonais au cours de leurs activités. La présence des malades, réservoir de parasites dans ce foyer historique, pourrait favoriser le réveil de la THA. En effet, le risque de transmission de cette dernière existe même dans des espaces où la pression humaine est faible, dès lors que les conditions environnementales favorisent la survie des glossines et que la mobilité humaine permet un contact étroit entre les hommes infectés (réservoir de parasites) et les vecteurs (Fournet *et al.*, 2001).

Afin de vérifier l’existence d’un risque trypanosomien potentiel dans le foyer historique de Bendjé, nous avons mené une enquête entomologique dans son épicentre d’alors, le canton Océan.

## Matériels et Méthodes

### Site d’étude

**Fig. 1.**
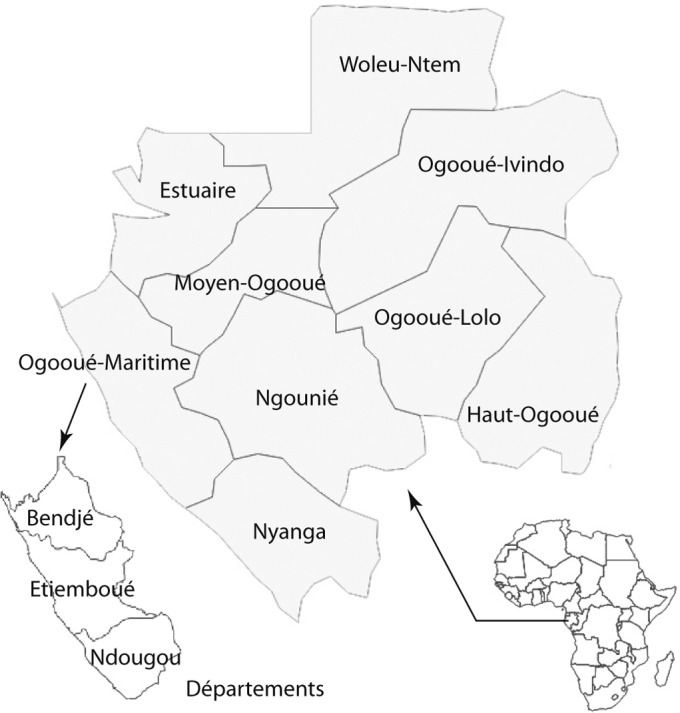
Le Gabon et le département de Bendjé dans la province de l’Ogooué-Maritime.

Bendjé est l’un des trois départements que compte la province de l’Ogooué-Maritime. Il est limité au nord et à l’ouest par l’océan Atlantique, au nord-est par les provinces de l’Estuaire et du Moyen-Ogooué et au sud par le département d’Etiemboué (Figure 1).

**Fig. 2.**
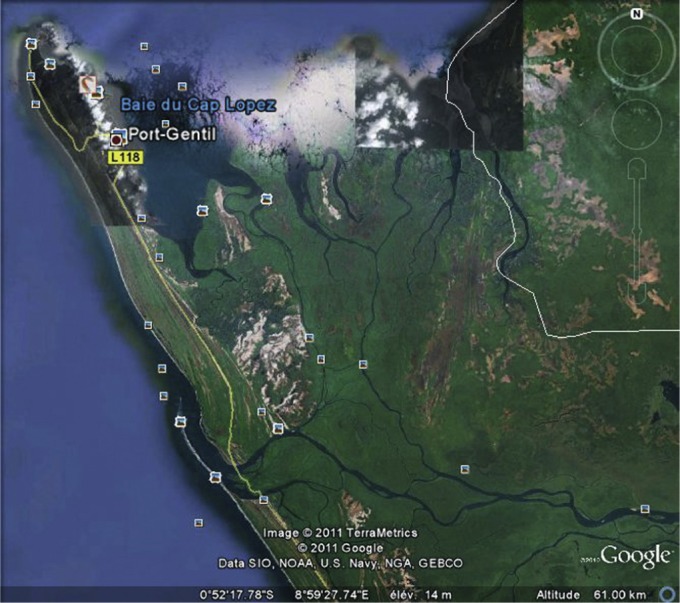
Image satellitaire de la zone d’étude (Google Earth – earth. google.com).

Cette étude a été conduite en 2009 dans le canton Océan qui a la particularité d’être situé à l’embouchure de nombreux cours d’eau qui délimitent des îlots avant de se jeter dans l’océan Atlantique (Figure 2). Ce canton est accessible uniquement par voie fluviale ou maritime.

Le climat est de type équatorial chaud et humide, caractérisé par deux saisons : une saison des pluies d’octobre à mai avec un léger fléchissement en janvier, une saison sèche de juin à la mi-septembre. Les températures moyennes annuelles oscillent autour de 26,5 °C (Saint-Vil, 1977).

**Fig. 3.**
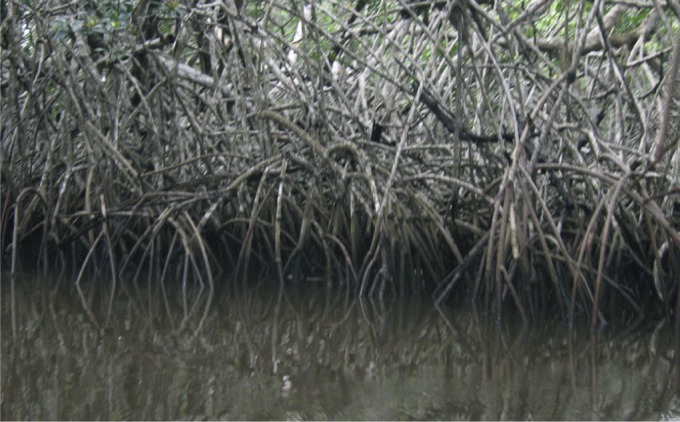
La mangrove dans le foyer de Bendjé, à marée basse.

La forêt dense sempervirente caractérise la végétation de la région. Dans la zone lagunaire, cette forêt est inondée et marécageuse ; elle se prolonge dans la zone de balancement des marées par une mangrove impénétrable riche en palétuviers blancs (*Rhizophora racemosa*) aux racines échasses (Figure 3).

Comme toutes les zones rurales de cette province du Gabon, le canton Océan est très peu peuplé; selon des informations recueillies sur place, les villages seraient peuplés uniquement en période de vacances scolaires. Le nombre d’habitants varie ainsi entre 50 et 300 par village. Au cours de notre étude, nous avons observé des villages d’une vingtaine de cases souvent habitées par un seul individu, conséquence de l’exode rural engendré par l’enclavement de la localité (absence d’établissements scolaires, de structures hospitalières...). L’habitat est de type groupé avec des cases disposées linéairement le long des cours d’eau. Il existe également des campements à habitation temporaire, bâtis sur des îlots et utilisés uniquement par des pêcheurs. La pêche constitue la principale activité de la région; elle est pratiquée aussi bien par des nationaux que par des étrangers originaires d’Afrique de l’Ouest (Bénin, Ghana, Cap Vert…).

Les cultures vivrières sont pratiquées à la lisière du village, derrière les cases, et se limitent à des champs de manioc et à des bananeraies. L’élevage est réduit à des animaux de compagnie (chat et chien) et à de la volaille.

### Prospection entomologique

Nous avons préalablement procédé à une exploration du milieu. Les informations concernant les populations et leurs habitudes quotidiennes ont été recueillies auprès du chef de village ou de toute autre notabilité présente; une visite guidée de l’environnement immédiat des villages, orientée vers les principaux lieux d’activités des populations, a aussi été effectuée. Le type de végétation de chacun des sites explorés a été noté et un circuit de piégeage des glossines a été tracé. Quatre biotopes ont été retenus en fonction du contexte écologique et du degré de fréquentation par les populations : débarcadères (lieux où accostent les pirogues), mangrove ou point de passage des pirogues, champs vivriers et points de baignade. Dans les îlots, seuls les débarcadères et les points de baignade ont été retenus comme sites de fréquentation des populations, les campements étant établis à moins de 50 mètres des débarcadères. Des pièges monoconiques (42 au total) de type Vavoua (Laveissière et Grébaut, 1990) ont été installés dans chacun de ces biotopes et ont été laissés en place pendant quatre jours. Dans la mangrove, le piège a été laissé en place uniquement en période de marée basse; il était posé tous les matins entre 9 et 10 h et retiré à 16 h. La collecte des glossines pour tous les pièges a été effectuée une fois par jour, à partir de 16 h en raison des difficultés de navigation à marée basse. Les glossines ont été dénombrées en fonction du sexe, puis identifiées sur la base des critères morphologiques (coloration des tarses et de l’abdomen) et de la structure des génitalia (Brunhes *et al.*, 1998; Pollock, 1982). Les glossines ténérales étaient préalablement identifiées par légère compression du thorax (sensation de souplesse entre le pouce et l’index) puis confirmées après dissection et observation d’un sac résiduel à l’extrémité postérieure de leur intestin antérieur (Laveissière, 1975). Les glossines ont été disséquées dans un délai de 24 h dans une goutte de solution saline (NaCl 9%) à la loupe binoculaire, et les pièces buccales, le tube digestif et les glandes salivaires observés au microscope pour la recherche des trypanosomes. Ces organes étaient ensuite collectés séparément dans des tubes contenant 50 μl d’éthanol à 70%, puis conservés dans de la glace sur le terrain (≈ 4 °C) et à - 20 °C au laboratoire pour des analyses ultérieures de biologie moléculaire en vue de l’identification des espèces de trypanosomes.

Lorsque l’intestin moyen contenait du sang frais ou du sang légèrement digéré, celui-ci a été prélevé, étalé sur du papier Whatman n° 1 et conservé dans un sac en plastique hermétiquement fermé et contenant du gel de silice. Ces repas de sang ont été ensuite analysés au laboratoire par la technique ELISA (Beier *et al.*, 1988) et les anticorps anti-homme, anti-porc, anti-cheval, anti-mouton, anti-poule, anti-chien et anti-boeuf ont été utilisés pour l’identification de l’origine du repas.

### Amplification de l’ADN

L’extraction d’ADN a été réalisée au Chelex 100 à 10%. Les échantillons broyés à l’aide d’un piston stérilisé ont été centrifugés à 14 000 tours/min pendant dix minutes. Au surnageant, était ajouté 100 μl de chelex 100 à 10% et les tubes incubés à 56 °C pendant une heure, puis à 95 °C pendant 30 minutes avant d’être centrifugés à nouveau pendant deux minutes. Le surnageant collecté a été conservé à - 20 °C pour amplification par PCR. Des couples d’amorces spécifiques aux espèces *Trypanosoma congolense* “type forêt” (TCF 1/2), *Trypanosoma brucei* s.l. (TBR 1/2) et *Trypanosoma vivax* (TVW 1/2) ont été utilisés (Masiga *et al.*, 1992; Morlais *et al.*, 1998). La réaction d’amplification a été réalisée dans un volume total de 25 μl contenant 1 μl d’ADN extrait, 1 μl de chacune des amorces testées, 0,25 unités de *Taq* (*Thermos Aquaticus*) DNA polymerase et 5 μl de tampon PCR-buffer enrichi de 45 mM Tris-HCl (pH 8,8), 11 mM (NH4)2SO4, 4,5 mM MgCl2, 6,7 mM 2-mercaptoéthanol, 4,4 μM EDTA, 113 μg/ml BSA, 1 mM de chacun des désoxynucléotides phosphate (dTTP, dATP, dCTP et dGTP); l’ADN génomique de chaque groupe taxonomique de trypanosome a été utilisé comme contrôle positif et de l’eau stérile pour le contrôle négatif. Une dénaturation à 94 °C pendant quatre minutes a précédé 30 cycles d’amplification comprenant chacune une dénaturation à 95 °C pendant une minute, une hybridation à 60 °C pendant une minute, une élongation à 65 °C pendant une minute puis une élongation finale à 72 °C pendant quatre minutes. Les réactions ont été visualisées sous ultraviolets sur gel d’agarose à 1,5% enrichi au SYBR®-*Safe DNA gel stain*. Les échantillons considérés positifs ont été ceux dont la reproductibilité des résultats a été obtenue au moins trois fois.

### Traitement des données

La densité apparente des glossines par jour (DAP) a été calculée en divisant l’effectif total de glossines capturées par le nombre de pièges posés dans le biotope considéré et par le nombre de jours de capture. Le taux d’infection des glossines a été exprimé par le pourcentage du nombre de glossines infestées sur le nombre total de glossines non ténérales disséquées.

Le contact homme-glossine (*p*) a été évalué en appliquant la relation définie par Laveissière *et al.* (1994) :

$$p=\frac{\left(\mathrm{kxnx}C^{a-1}\right)}{Pj^{a}}$$

où *k* et *a* sont des constantes, *n* le nombre de repas de sang d’origine humaine identifié, *C* le nombre total de glossines disséquées, *P* le nombre de pièges utilisés et *j* le nombre jours de capture. En Côte d’Ivoire, il a été démontré que les constantes *k* et *a* étaient égales à 632 et 1,23 respectivement dans les plantations de café et de cacao et à 623 et 0,63 respectivement à la lisière des cases (Laveissière *et al.*, 1994). Aussi, dans cette étude, nous avons considéré que la constante *k* est égale à 623 dans les champs vivriers (du fait de leur localisation à la lisière des cases), à 632 dans la mangrove et au niveau des débarcadères; la constante *a* a été fixée à 0,63 dans les champs vivriers et à 1,23 en zone de mangrove et au niveau des débarcadères.

L’indice du risque épidémiologique dans chacun des biotopes prospectés a été calculé selon la formule suivante (Laveissière et Grébaut, 2003) :

$$r=\frac{(t+1)xn^{2}xC^{0,46}}{Pj^{3,69}}$$

*t* désignant le nombre de glossines ténérales capturées, *n* le nombre de repas de sang d’origine humaine, *C* le nombre total de glossines capturées, *P* le nombre de pièges et *j* le nombre de jours de capture.

## Résultats

### Distribution des glossines et taux d’infection

Au total, 17 villages et campements ont été prospectés dont 13 localisés sur la partie continentale et quatre bâtis sur des îlots. Les 1 544 glossines capturées ont été identifiées comme appartenant aux taxons *Glossina palpalis palpalis* Robineau-Desvoidy, 1830 (99,03%), *Glossina pallicera newsteadi* Austen, 1929 (0,78%) et *Glossina caliginea* Austen, 1911 (0,19%). *G. palpalis palpalis* a été capturée dans tous les types de biotopes explorés, aussi bien dans la partie continentale que sur les îlots; *G. pallicera newsteadi* a été capturée dans les champs vivriers et uniquement sur le continent, et *G. caliginea* au niveau des débarcadères du continent et des îlots. La densité apparente des glossines par piège et par jour variait d’un type de biotope à un autre; des densités élevées de glossines ont été observées au niveau des débarcadères et des champs vivriers du continent et au niveau des points de baignade des îlots (Tableau I). Aucune glossine ténérale n’a été capturée dans la mangrove.

**Tableau I.** Paramètres entomologiques de la transmission dans les différents biotopes du foyer de Bendjé.

	Débarcadères	Mangrove	Champs vivriers	Point de baignade
DAP	8,68^a^/3,32^b^	3,80^a^/0,00^b^	6,22^a^/0,00^b^	3,64^a^/4,00^b^
T	1,25^a^/0^b^	0,00^a^	0,66^a^	1,00^a^/0^b^
T.I. (%)	19,35^a^/0^b^	2,56 a	5,56^a^	8,86^a^/0^b^
*n*	3,00^a^/0^b^	1,00^a^	2,00^a^	3,00^a^/0^b^
*p*	385,13^a^/0^b^	220,39^a^	l65,78^a^	l47,40^a^/0^b^
*r*	0,00020^a^/0^b^	0,00^a^	0,000l4^a^	0,000070^a^/0^b^

a partie continentale ;

b îlots ; DAP : densité apparente des glossines par piège et par jour; T : densité apparente des glossines ténérales; T.I. : taux d’infection; n : nombre de repas de sang d’origine humaine; p : point de contact homme-glossine; r : index du risque de contamination

Seules 268 glossines ont été disséquées; les autres ayant séchées du fait de l’exposition prolongée au soleil, consécutive au relevé irrégulier de pièges (une seule fois par jour). Des glossines parasitées ont été retrouvées dans tous les biotopes. Un taux d’infection global de 39,92% (107 glossines infectées/268 disséquées) a été obtenu après dissection des glossines; ce taux variait de 5,13 à 50% par village. Les infections ont été observées à majorité au niveau du proboscis seul (47,66%; 51/107) et uniquement chez *G. palpalis palpalis*, puis dans l’intestin seul (29,90%) à la fois chez *G. palpalis palpalis* (30 glossines infectées/105 glossines disséquées de cette espèce) et chez *G. pallicera newsteadi* (2 glossines infectées/2 glossines disséquées de cette espèce). Les infections du proboscis et de l’intestin ont été également observées chez *G. palpalis palpalis* (22,43%; 24 glossines infectées/107 glossines disséquées) et aucune infection de glande salivaire n’a été observée. De même, aucune infection n’a été observée chez *G. caliginea*.

Les organes microscopiquement infectés ont été prioritairement analysés par PCR et nous n’avons pris en compte que les résultats ayant une positivité répétitive minimale de trois fois. Un taux d’infestation global par PCR de 22,39% (60 glossines positives par PCR /268 disséquées) a été obtenu et deux espèces de trypanosomes ont été identifiées : *Trypanosoma vivax* (39/60 glossines positives par PCR) et *T. brucei* s.l. (21/60 glossines positives par PCR). Ce taux d’infection est variable suivant les biotopes et des taux élevés ont été observés au niveau des débarcadères et des points de baignade (Tableau I) avec une différence significative entre ces deux biotopes (χ^2^ = 9,42; ddl = 1; p < 0,01). Ces parasites ont été retrouvés chez les sous-espèces *G. palpalis palpalis* (58 glossines) et *G. pallicera newsteadi* (2 glossines). Aucune glossine infestée n’a été retrouvée dans les îlots.

### Localisation du contact homme-glossines et indice du risque de transmission

Parmi les 18 repas de sang testés, neuf ont été d’origine humaine dont deux repas mixtes, homme-chien et homme-poule; tous ces repas ont été prélevés sur des glossines capturées sur le continent. L’origine des autres repas n’a pu être identifiée avec les anticorps utilisés. Un contact homme-glossine a été ainsi objectivé dans tous les types de biotopes prospectés du continent, le contact le plus intense se situant au niveau des débarcadères. Cependant, le risque de transmission est très inférieur à 1 dans la plupart des biotopes prospectés et nul dans la mangrove (Tableau I).

## Discussion

L’abondance de *G. palpalis palpalis* dans ce foyer concorde avec l’ancienne carte de distribution des glossines au Gabon (Maillot, 1953) qui montre une large distribution de cette sous-espèce dans la partie du territoire infestée de glossines. Son ubiquité dans tous les biotopes prospectés témoigne de son affinité pour l’humidité relative et les températures comprises entre 20 et 25 °C, favorisées ici par la présence de l’eau qui constitue avec la végétation les facteurs environnementaux idéals pour sa survie (Schwetz, 1915). La source de nourriture est également un facteur qui explique la distribution de *G. palpalis palpalis* dont l’éclectisme alimentaire favorise l’adaptation à différents milieux écologiques (Baldry, 1980; Laveissière *et al.*, 2000). *G. pallicera newsteadi* a été retrouvée uniquement dans les milieux hautement anthropisés que sont les champs vivriers situés à la lisière des cases et *G. caliginea* uniquement dans les débarcadères : cette espèce est connue pour sa prédilection pour les marécages d’eau douce près du littoral et les zones de forêt à mangrove (Leak, 1999). Aucune espèce du groupe *fusca* n’a été capturée, connue pourtant comme des espèces des zones de forêt humide (Jordan, 1963). L’absence des espèces de ce groupe dans l’échantillonnage capturé serait due au circuit de piégeage qui n’a concerné que des biotopes anthropisés; il existe une relation inverse entre la densité des populations humaines et la présence des glossines du groupe *fusca* (Jordan 1962). Tous les biotopes prospectés – exceptée la mangrove où des inondations fréquentes liées aux fluctuations de la marée empêchent la maturation et l’éclosion des pupes – constituent des gîtes permanents de glossines.

La présence des glossines infectées et l’identification des trypanosomes animaux témoignent de l’existence d’un risque trypanosomien animal et suggère l’établissement d’un cycle de transmission animal-glossineanimal dans le foyer. *T. vivax* est l’espèce prédominante dans le foyer; une étude similaire, réalisée en 1977, et basée uniquement sur la dissection des glossines, y avait déjà mis en évidence la présence de cette espèce (Eouzan, 1977). Il a été signalé une corrélation entre les infections à *T. vivax* et les repas pris sur bovidés (Jordan, 1965); aucun repas de sang de glossine testé ne provenait de cette famille de vertébrés, inexistante dans la localité. L’absence de glande salivaire infectée suggère *a priori* l’absence de la sous-espèce de trypanosome humain dans le foyer. Sachant que des individus originaires du foyer et parasitologiquement positifs sont passivement diagnostiqués en deuxième phase de la maladie du sommeil, il est assez difficile d’admettre pareille hypothèse. Davantage d’analyses de biologie moléculaire nous permettraient d’élucider la question.

Très peu de glossines gorgées (1,17%) ont été capturées. Ce résultat est similaire à ceux de Dagnogo *et al.* (1985) en Côte d’Ivoire et de Simo *et al.* (2008) au Cameroun qui ont obtenus respectivement 3 et 2,8% de repas de sang de glossines au sein de leur échantillon après capture au piège. La très faible proportion de glossines gorgées capturées au piège s’expliquerait par le fait que les glossines passent la plupart de leur temps au repos pendant lequel elles digèrent leur repas. Ceci avait été observé en secteur pré-forestier de Côte d’Ivoire par Gouteux *et al.* (1984) qui ont obtenu 30% de mouches gorgées sur un échantillonnage de glossines capturées dans les lieux de repos. Après son repas, la glossine s’envole aussitôt pour se poser dans un endroit où elle pourra excréter, pendant quelques minutes, l’excédent d’eau. Il est probable que les conditions climatiques de ce lieu de repos temporaire l’obligent à rechercher ensuite un lieu de repos plus favorable où elle pourra bien digérer son repas. Ce serait donc probablement pendant le vol entre ces deux lieux que les glossines gorgées capturées par piégeage sont interceptées (Dagnogo *et al.*, 1985).

La moitié des repas de sang de glossines testés était d’origine humaine; cette information mérite d’être à nouveau vérifiée eu égard au biais de la technique de capture utilisée au cours de notre étude. Toutefois, la présence de l’homme dans le foyer comme hôte nourricier potentiel de la tsé-tsé soulève davantage la question de l’existence de la sous-espèce de trypanosome humain qui nécessite d’être clarifiée.

Aucun contact entre l’homme et les glossines n’a été retrouvé aux niveaux des îlots; en revanche, un contact a été retrouvé dans tous les faciès écologiques de la partie continentale du foyer. Le comportement humain expliquerait l’absence de contact entre les glossines et l’homme au niveau des îlots. Ce sont des campements temporaires de pêcheurs utilisés essentiellement lors des repos nocturnes, or les glossines sont des insectes à activité diurne. L’anthropophilie de ces insectes dépend de la concurrence des animaux hôtes (Hervouet et Laveissière, 1987) et il a été rapporté expérimentalement que la glossine aurait tendance à se nourrir sur la même espèce hôte que celle de son premier repas (Bouyer *et al.*, 2005).

Un fort pourcentage de repas de sang pris sur l’homme n’est pas le signe d’une transmission intense de la maladie du sommeil. Malgré la présence d’un contact permanent entre l’homme et les glossines dans la partie continentale du foyer, l’indice de risque épidémiologique est nul dans la mangrove et très faible dans les autres biotopes. Le foyer de Bendjé est une localité très peu peuplée où la présence de l’homme reste transitoire. Sachant que le risque de transmission de la maladie du sommeil est d’autant plus grand que la présence de l’homme dans l’aire de distribution de la glossine est durable (Laveissière *et al.*, 1985), on pourrait dire que la probabilité d’une endémisation de la maladie du sommeil dans le foyer de Bendjé reste très faible. Cependant, la proximité de l’homme et de la glossine dénote l’existence d’une situation épidémiologiquement dangereuse si le rapprochement entre l’homme et le vecteur tend à se maintenir, et surtout si le parasite humain (dont la prévalence dans le foyer reste à démontrer) est introduit dans le milieu par un individu malade ou une glossine infectée.
